# Genetic associations between the miRNA polymorphisms miR-130b (rs373001), miR-200b (rs7549819), and miR-495 (rs2281611) and colorectal cancer susceptibility

**DOI:** 10.1186/s12885-019-5641-1

**Published:** 2019-05-22

**Authors:** Eun-Gyo Kim, Jung Oh Kim, Han Sung Park, Chang Soo Ryu, Jisu Oh, Hak Hoon Jun, Jong Woo Kim, Nam Keun Kim

**Affiliations:** 1 Department of Biomedical Science, College of Life Science, CHA University, 335 Pangyo-ro, Bundang-gu, Seongnam, 13488 South Korea; 2 Department of Internal Medicine, CHA Bundang Medical Center, CHA University, 59 Yatap-ro, Bundang-gu, Seongnam, 13496 South Korea; 3 Department of Surgery, CHA Bundang Medical Center, CHA University, 59 Yatap-ro, Bundang-gu, Seongnam, 13496 South Korea

## Abstract

**Background:**

Recent studies have extensively investigated the role of miRNAs in colorectal cancer (CRC), and several associations have been reported. In addition, single nucleotide polymorphisms (SNPs) in promoter regions of miRNAs have been shown to affect miRNA expression. Therefore, we aimed to analyze the effect of miRNA polymorphisms on CRC susceptibility.

**Methods:**

We conducted association studies on the relationships between the miRNA polymorphisms *miR-130b*T > C rs373001, *miR-200b*T > C rs7549819, and miR-495A > C rs2281611 and CRC with 472 CRC patients and 399 control subjects in Korea.

**Results:**

Multivariate logistic regressions of the CRC subgroups showed that the *miR-495*CC genotype associated with rectal cancer (AA+AC vs. CC; adjusted odds ratio (AOR) for CC, 1.592; 95% confidence interval (CI), 1.071–2.368; *P* = 0.022). The gene-environment combinatorial analysis showed that the combination of *miR-495*A > C and low plasma folate contributed to an increased risk of rectal cancer (AA+AC vs. CC; AOR for CC, 3.829; 95% CI, 1.577–9.300; *P* = 0.003). In the survival analysis, *miR-200b*T > C associated with CRC patient mortality (TT vs TC + CC; adjusted hazard ratio for TC + CC, 0.592; 95% CI, 0.373**–**0.940; *P* = 0.026).

**Conclusion:**

In this study, we found that *miR-200b* and miR*-495* polymorphisms are involved in CRC susceptibility and prognosis.

**Electronic supplementary material:**

The online version of this article (10.1186/s12885-019-5641-1) contains supplementary material, which is available to authorized users.

## Background

Colorectal cancer (CRC) is the third most prevalent cancer in the world with a high mortality rate [1], and eating habits and lifestyle patterns contribute to the high incidence in developed countries [2]. However, studies on dietary habits and lifestyle patterns have failed to sufficiently explain CRC disease outbreaks. Many groups have therefore focused on identifying the genetic causes of CRC, and molecular mechanisms such as microsatellite instability (MSI), CpG island methylator phenotype (CIMP), chromosomal instability (CIN), and *KRAS* or *BRAF* mutations have been described [3–7]. Recent studies indicate that microRNAs are potential prognostic biomarkers of CRC [8, 9].

MicroRNAs (miRNAs, miR) are small RNAs of ~ 22 bases, which bind to 3′-untranslated regions (UTRs) of target mRNAs to post-transcriptionally regulate the corresponding genes by silencing or degrading the mRNAs [10–12]. miRNAs are involved in many biochemical and metabolic pathways in many organisms, and most miRNAs exist in the noncoding regions of genes [13]. miRNA is firstly transcribed into primary miRNA (pri-miRNA) and then transformed into precursor miRNA (pre-miRNA) by the DGCR8-DROSHA complex. Pre-miRNA is transported to the cytoplasm by the RAN-GTP/exportin-5 complex, where it is processed into a mature miRNA by DICER. Mature miRNA functions in an RNA-induced silencing complex (RISC) complex that targets mRNA [14]. Previous studies have revealed associations between miRNA expression and various cancers, including leukemia [15], hepatocarcinoma [16], gastric cancer [17], bladder cancer [18], lung cancer [19], and breast cancer [20]. It has also been shown that polymorphisms in miRNA sequences regulate miRNA expression [21, 22]. Studies have confirmed associations between miRNA polymorphisms and cancer development, progression, and metastasis [23–25].

We previously demonstrated that *miR-146a*, *miR-149*, *miR-196a2*, and *miR-499* single nucleotide polymorphisms (SNPs) associate with CRC [26]. However, because additional miRNA polymorphisms may associate with CRC, we asked whether *miR-130b*, *miR-200b*, and *miR-495* SNPs also associate with CRC. *MiR-130b* has been shown to contribute to the occurrence of CRC and is involved in the PTEN/AKT signaling pathway [27, 28]. In addition, *miR-200b* has been shown to affect the breast cancer survival rate [29], to be involved in the regulation of c-Myc/PRDX2 in CRC [30], and to affect the migration, invasion, and epithelial mesenchymal transition (EMT) mechanisms of lung cancer [31]. *miR-495* has been reported to reduce the proliferation of cancer cells in CRC and breast cancer [32, 33] and to affect cancer metastasis [34].

As mentioned earlier, *miR-130b*, *200b*, and *495* have been linked to CRC development and progression. We focused on three SNPs: *miR-130b* rs373001T > C, *miR-200b* rs7549819T > C, and *miR-495* rs2281611A > C, all of which are regulatory regions of miRNA expression. We hypothesized that polymorphisms in these miRNAs would ultimately influence CRC susceptibility and mortality. There is no known genetic association of these SNPs with CRC. This study specifically examined whether miRNA polymorphisms are related to CRC susceptibility in Koreans.

## Methods

### Study population

For this case-control study, a total of 871 individuals were enrolled from June 2005 to January 2011, including 472 patients diagnosed with CRC at CHA Bundang Medical Center (Seongnam, South Korea) and 399 randomly selected non-CRC subjects who participated in a health-screening program. This case group included only CRC patients who had gone through surgery and who had confirmed to adenocarcinoma by histology. The case group included colon and rectal cancer patients (268 and 193 patients, respectively). Tumors were classified by their tumor, node and metastasis classification (TNM) stage according to the 7th of the American joint committee on cancer (AJCC) staging manual as follows: stage I, *n* = 52 (11.02%); stage II, *n* = 191 (40.47%); stage III, *n* = 176 (37.29%); and stage IV, *n* = 47 (9.96%). Hypertension (HTN) and diabetes mellitus (DM) for overall participants were classified according to the criteria of the previous study [35]. We had were provided written informed consent for all of the participants and the study protocol was approved by the Institutional Review Board of CHA Bundang Medical Center (IRB No. 2009–08-077) and followed the recommendations of the Declaration of Helsinki.

### Genotyping

DNA was extracted from white blood cells using a “G-DEX™IIb For Blood kit” (iNtRON Biotechnology, South Korea). Genotyping of *miR-130b* rs373001T > C, *miR-200b* rs7549819T > C and *miR-495* rs2281611A > C were performed by same protocol as in our previous study [36], and detailed PCR conditions were presented in Additional file 1: Table S1. We randomly repeated 10–15% of *miR-130b* rs373001T > C, *miR-200b* rs7549819T > C and *miR-495* rs2281611A > C polymorphism genotyping results and confirmed the results with DNA sequencing [36]. The concordance between the experiment and randomly repeat was 100%.

### Statistical analysis

To compare clinical characteristics between study groups, we used the χ^2^ test and the two-tail *t*-test or Mann-Whitney test. The adjusted odds ratios (AORs) and 95% confidence intervals (CIs) for association with miRNAs polymorphisms in CRC risk were calculated by multivariate logistic regression adjusted for age, sex, HTN, and DM. The software program used for statistical analysis in this study were “GraphPad Prism 4.0” (GraphPad Software Inc., San Diego, CA, USA), “HAPSTAT 3.0” (University of North Carolina, Chapel Hill, NC, USA), and “Medcalc v.18.2.1” (Medcalc Software, Mariakerke, Belgium) and and the cut-off of statistically significant was considered was *P* values < 0.05. The false discovery rate (FDR) was calculated when performing multiple comparisons to estimate the overall experimental error rate resulting from false positives. Independent prognostic markers were investigated using the Cox proportional-hazards regression for mortality analysis, and the results were adjusted for age, sex, TNM stage, and chemotherapy. Hazard ratios (HRs) are shown with 95% CIs.

## Results

### Study subject characteristics

The 472 CRC cases included 212 males and 260 females with an overall mean age of 61.99 ± 12.32 years. There were no significant differences in the age and sex of the CRC patients and the controls (*P* = 0.290 and 0.774, respectively). The baseline characteristics of patients with colon and rectal cancers, which are subgroups of CRC, showed no statistical differences when compared to the control group **(**Table 1**)**.

**Table 1:** Baseline characteristics between controls and CRC patients

Characteristic	Controls (*n* = 399)	CRC Patients (*n* = 472)	*P*	Colon cancer (*n* = 268)	*P*	Rectal cancer (*n* = 193)	*P*
Age (years, mean ± SD)	61.15 ± 10.93	61.99 ± 12.32	0.129	61.44 ± 12.88	0.464	62.28 ± 11.54	0.153
Male (%)	173 (43.4)	212 (44.9)	0.645	118 (44.0)	0.915	88 (45.6)	0.750
Hypertension (%)	155 (38.8)	281 (59.5)	< 0.0001	157 (58.6)	0.003	117 (60.6)	0.003
HDL-C (mg/dL, mean ± SD)	45.91 ± 13.48	42.18 ± 13.05	0.001	42.82 ± 13.00	0.013	41.27 ± 13.07	0.001
LDL-C (mg/dL, mean ± SD)	115.87 ± 40.28	101.31 ± 28.62	0.003	98.55 ± 28.01	0.002	104.32 ± 29.54	0.142
Diabetes mellitus (%)	52 (13.0)	156 (33.1)	< 0.0001	92 (34.3)	< 0.0001	64 (33.2)	< 0.0001
Smoking (%)	138 (34.6)	92 (19.5)	< 0.0001	55 (20.5)	0.003	35 (18.1)	0.002
Folate (nmol/L, mean ± SD)	8.64 ± 6.13	7.94 ± 7.13	< 0.0001	8.12 ± 7.36	0.001	7.70 ± 6.86	0.000
Triglyceride (mg/dL, mean ± SD)	146.79 ± 89.33	129.00 ± 86.30	0.0003	126.93 ± 84.48	0.001	132.48 ± 90.86	0.015
Homocysteine (μmol/L, mean ± SD)	9.96 ± 4.27	10.68 ± 7.83	0.671	10.47 ± 8.21	0.572	10.88 ± 7.32	0.215
Total cholesterol (mg/dL, mean ± SD)	192.00 ± 37.32	178.76 ± 40.56	0.0001	178.73 ± 38.88	0.001	176.69 ± 42.89	0.002
Tumor size (%)							
< 5 cm		208 (44.1)		106 (39.6)		93 (48.2)	
≥ 5 cm		264 (55.9)		162 (60.4)		100 (51.8)	
TNM stage (%)							
I		52 (11.2)		26 (9.7)		26 (13.5)	
II		191 (41.0)		118 (44.2)		70 (36.3)	
III		176 (37.8)		94 (35.2)		81 (42.0)	
IV		47 (10.1)		29 (10.9)		16 (8.3)	
N.A.		6		1		0	
MSI (%)		61 (15.6)		49 (22.0)		12 (7.3)	
MSI-high (%)		46 (11.8)		38 (17.0)		8 (4.9)	
MSI-low (%)		15 (3.8)		11 (4.9)		4 (2.4)	
N.A.		82		45		29	

*P-values* were calculated by Man whithney *U* test for continuous variables and chi-square test for categorical variables.TNM stage, TNM classification of malignant tumours; *MSI*, microsatellite instability; N.A. row, missing data

### Genotype frequencies

The distributions of genotypes for the miRNA polymorphisms *miR-130b*T > C, *miR-200b*T > C, and *miR-495*A > C in CRC patients and control subjects are shown in Table 2**.** The genotype frequencies of CRC and control groups were in Hardy-Weinberg equilibrium (HWE). There was no statistically significant difference in the distribution of *miR-130b*T > C, *miR-200b*T > C, and *miR-495*A > C SNPs between the CRC and control groups. In a subgroup analysis, we observed that the *miR-495*CC genotype was more frequent in rectal cancer patients than in the control group (AA+AC vs. CC; AOR for CC, 1.592; 95% CI, 1.071–2.368; Table 3). However, this statistical significance was lost after correcting for multiple comparisons using the FDR method (*P* = 0.065). There were no statistically significant differences in the distributions of the other miRNA SNPs between the CRC subgroups and the control group. We also confirmed that these SNPs are not associated to the MSI status (Additional file 1: Table S2).

**Table 2:** Genotype frequencies of microRNA polymorphisms in CRC patients and control subjects

Genotypes	Controls(*n* = 399)	Patients(*n* = 472)	AOR (95% CI)	*P*	FDR-*P*
*miR-130b* rs373001T > C					
TT	216 (54.2)	269 (57.0)	1.000 (reference)		
TC	157 (39.3)	168 (35.6)	0.825 (0.610–1.115)	0.210	0.416
CC	26 (6.5)	35 (7.4)	0.943 (0.532–1.670)	0.840	0.840
Dominant (TT vs TC + CC)			0.846 (0.635–1.127)	0.254	0.398
Recessive (TT + TC vs CC)			1.028 (0.590–1.792)	0.923	0.923
HWE *P*	0.723	0.222			
*miR-200b* rs7549819T > C					
TT	171 (42.9)	216 (45.7)	1.000 (reference)		
TC	176 (44.1)	200 (42.4)	0.882 (0.652–1.194)	0.416	0.416
CC	52 (13.0)	56 (11.9)	0.758 (0.481–1.194)	0.232	0.696
Dominant (TT vs TC + CC)			0.850 (0.638–1.132)	0.266	0.398
Recessive (TT + TC vs CC)			0.789 (0.512–1.215)	0.281	0.422
HWE *P*	0.527	0.356			
*miR-495* rs2281611A > C					
AA	103 (25.8)	125 (26.5)	1.000 (reference)		
AC	194 (48.6)	222 (47.0)	0.829 (0.584–1.176)	0.292	0.416
CC	102 (25.6)	125 (26.5)	1.080 (0.734–1.590)	0.696	0.840
Dominant (AA vs AC + CC)			0.919 (0.666–1.268)	0.608	0.608
Recessive (AA+AC vs CC)			1.208 (0.897–1.626)	0.214	0.422
HWE *P*	0.582	0.197			

*AOR*, adjusted odds ratio (adjusted for age, gender, hypertension, diabetes mellitus); *CI*, confidence interval; *FDR*, false discovery ratio; *HWE*, Hardy-Weinberg equilibrium

**Table 3:** Genotype frequencies of microRNA polymorphisms in CRC subgroups and control subjects

Genotypes	Controls(*n* = 399)	Colon(*n* = 268)	AOR (95% CI)	*P*	FDR-*P*	Rectal(*n* = 193)	AOR (95% CI)	*P*	FDR-*P*
*miR-130b* rs373001T > C									
TT	216 (54.2)	156 (58.2)	1.000 (reference)			109 (56.5)	1.000 (reference)		
TC	157 (39.3)	97 (36.2)	0.830 (0.585–1.177)	0.295	0.443	68 (35.2)	0.812 (0.549–1.201)	0.298	0.446
CC	26 (6.5)	15 (5.6)	0.671 (0.327–1.377)	0.276	0.717	16 (8.3)	1.061 (0.520–2.164)	0.871	0.871
Dominant (TT vs TC + CC)			0.805 (0.575–1.126)	0.205	0.371		0.858 (0.593–1.241)	0.415	0.908
Recessive (TT + TC vs CC)			0.740 (0.369–1.486)	0.398	0.909		1.166 (0.583–2.331)	0.664	0.664
*miR-200b* rs7549819T > C									
TT	171 (42.9)	126 (47.0)	1.000 (reference)			83 (43.0)	1.000 (reference)		
TC	176 (44.1)	109 (40.7)	0.826 (0.580–1.177)	0.290	0.443	88 (45.6)	1.091 (0.740–1.608)	0.660	0.660
CC	52 (13.0)	33 (12.3)	0.835 (0.493–1.412)	0.500	0.717	22 (11.4)	0.817 (0.449–1.488)	0.509	0.764
Dominant (TT vs TC + CC)			0.822 (0.589–1.146)	0.248	0.371		1.022 (0.706–1.480)	0.908	0.908
Recessive (TT + TC vs CC)			0.876 (0.531–1.447)	0.606	0.909		0.775 (0.441–1.362)	0.375	0.563
*miR-495* rs2281611A > C									
AA	103 (25.8)	72 (26.9)	1.000 (reference)			51 (26.4)	1.000 (reference)		
AC	194 (48.6)	135 (50.4)	0.881 (0.587–1.321)	0.540	0.540	78 (40.4)	0.744 (0.470–1.176)	0.205	0.446
CC	102 (25.6)	61 (22.8)	0.919 (0.583–1.450)	0.717	0.717	64 (33.2)	1.319 (0.810–2.147)	0.265	0.764
Dominant (AA vs AC + CC)			0.900 (0.618–1.310)	0.581	0.581		0.940 (0.621–1.421)	0.768	0.908
Recessive (AA+AC vs CC)			0.991 (0.675–1.453)	0.961	0.961		1.592 (1.071–2.368)	0.022	0.065

*CRC*, colorectal cancer; *AOR*, adjusted odds ratio (adjusted for age, gender, hypertension, diabetes mellitus); *CI*, confidence interval; *FDR*, false discovery ratio; *HWE*, Hardy-Weinberg equilibrium

### Combinatorial effects of miRNA polymorphisms and environmental factors

Because CRC has been shown to be influenced by various environmental factors, we performed a stratified analysis of age, sex, HTN, DM, and test levels of peripheral blood factors (homocysteine, folate, TG, HDL) to determine whether there was an association between miRNA polymorphisms and CRC risk (Additional file 1: Table S3). We did not find any associations between miRNA polymorphisms and CRC risk in the high-risk groups for each variable.

**Fig. 1:**
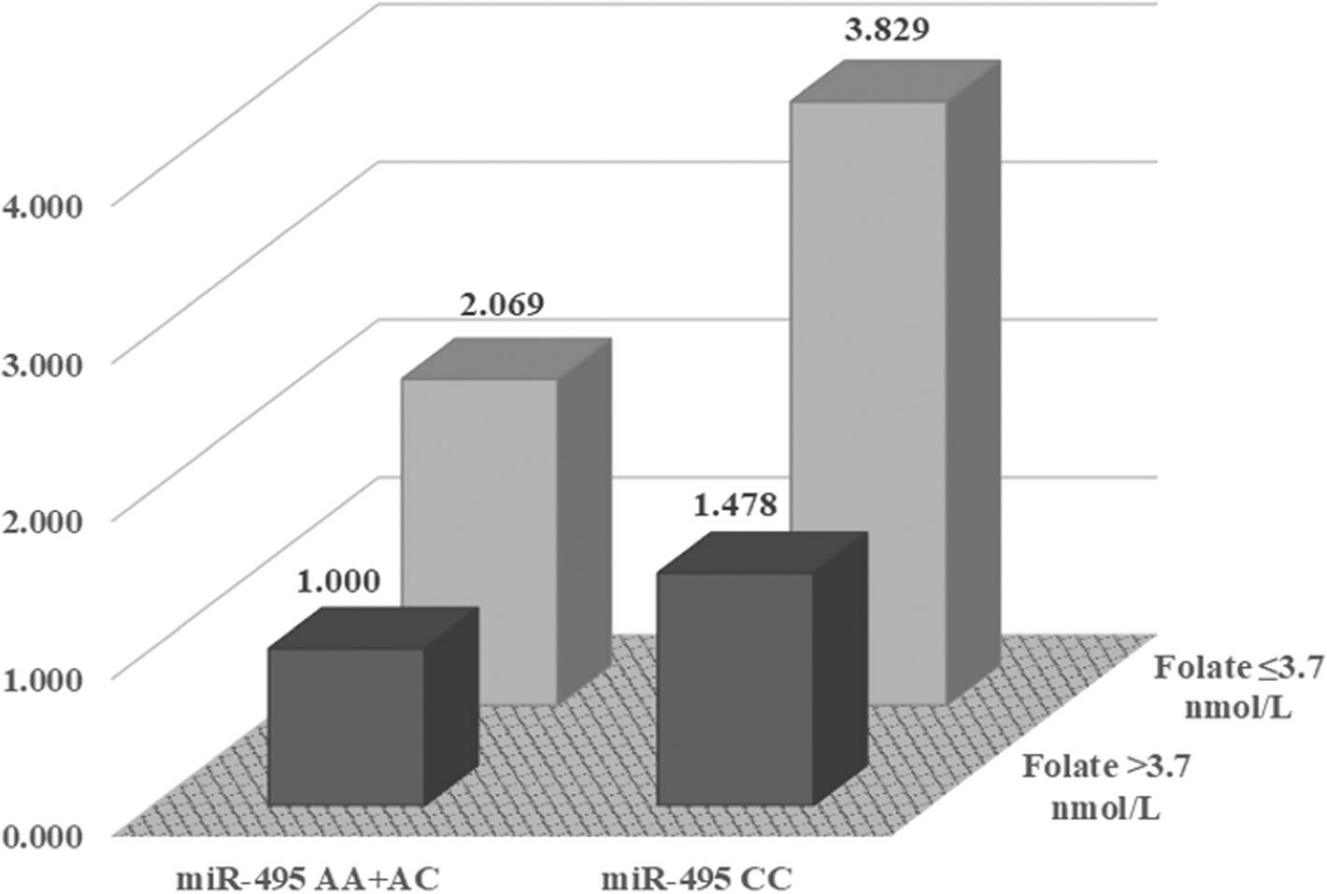
Combinatorial effect of *miR-495*C > A and folic acid on rectal cancer. Each row represents low or high plasma folate levels. Folate was divided into two concentration groups by eliminating the lower 15%. The columns represent the *miR-495*AA + AC and the *miR-495*CC genotypes. The y-axis represents the odds ratio for each group based on the reference group

We then conducted a gene-environment analysis to assess the combined effects of *miR-130b*T > C, *miR-200b*T > C, or *miR-495*A > C polymorphisms and clinical factors on CRC and CRC subgroup susceptibility. The combination of *miR-495*A > C and low plasma folate level contributed to an increased risk for CRC (AA+AC vs. CC; AOR, 3.119; 95% CI, 1.432–6.791; Additional file 1: Table S4). In addition, the *miR-495*CC genotype exhibited an increased risk in rectal cancer patients with HTN (AOR, 3.404; 95% CI, 1.902–6.092, *P* < 0.001), DM (AOR, 3.758; 95% CI, 1.685–8.383; *P* = 0.001), and in rectal cancer patients with low plasma folate levels (AOR, 3.829; 95% CI, 1.577–9.300; *P* = 0.003 Table 4 and Fig. 1).

**Table 4:** Combinatorial effects of miRNA polymorphisms and environmental factors on rectal cancer risk

Characteristics	*miR-130b*TT	*miR-130b*TC + CC	*miR-200b*TT	*miR-200b*TC + CC	*miR-495*AA + AC	*miR-495*CC
	AOR (95% CI)	AOR (95% CI)	AOR (95% CI)	AOR (95% CI)	AOR (95% CI)	AOR (95% CI)
Age						
< 63 years	1.000 (reference)	1.222 (0.706–2.115)	1.000 (reference)	1.032 (0.599–1.780)	1.000 (reference)	1.563 (0.874–2.793)
≥ 63 years	1.097 (0.672–1.790)	0.696 (0.412–1.176)	0.826 (0.475–1.436)	0.854 (0.515–1.415)	1.107 (0.706–1.736)	1.784 (1.038–3.064)
Gender						
Male	1.000 (reference)	0.799 (0.469–1.362)	1.000 (reference)	1.214 (0.707–2.085)	1.000 (reference)	1.772 (0.975–3.221)
Female	1.087 (0.664–1.782)	0.966 (0.570–1.636)	1.454 (0.835–2.531)	1.303 (0.753–2.255)	0.885 (0.565–1.387)	1.273 (0.730–2.222)
Hypertension						
No	1.000 (reference)	0.924 (0.531–1.610)	1.000 (reference)	0.799 (0.457–1.399)	1.000 (reference)	1.906 (1.050–3.461)
Yes	2.539 (1.535–4.200)	1.854 (1.076–3.196)	1.921 (1.101–3.350)	2.171 (1.279–3.683)	2.362 (1.496–3.727)	3.404 (1.902–6.092)
Diabetes mellitus						
No	1.000 (reference)	0.832 (0.544–1.274)	1.000 (reference)	0.872 (0.571–1.332)	1.000 (reference)	1.686 (1.077–2.642)
Yes	2.535 (1.382–4.651)	2.545 (1.385–4.676)	1.946 (0.998–3.793)	3.261 (1.798–5.913)	3.088 (1.851–5.152)	3.758 (1.685–8.383)
Homocysteine (μmol/L)						
< 13.3	1.000 (reference)	0.795 (0.531–1.191)	1.000 (reference)	1.191 (0.794–1.787)	1.000 (reference)	1.641 (1.069–2.518)
≥ 13.3	0.936 (0.451–1.943)	1.199 (0.579–2.484)	1.938 (0.904–4.152)	0.820 (0.394–1.708)	1.211 (0.653–2.248)	1.619 (0.612–4.282)
Folate (nmol/L)						
> 3.7	1.000 (reference)	0.853 (0.571–1.272)	1.000 (reference)	0.977 (0.654–1.458)	1.000 (reference)	1.478 (0.956–2.286)
≤ 3.7	2.427 (1.152–5.114)	2.193 (0.948–5.076)	1.645 (0.685–3.953)	2.512 (1.228–5.138)	2.069 (1.016–4.216)	3.829 (1.577–9.300)
Triglyceride (mg/dL)						
< 150	1.000 (reference)	0.843 (0.545–1.303)	1.000 (reference)	0.902 (0.584–1.394)	1.000 (reference)	0.934 (0.567–1.538)
≥ 150	0.524 (0.300–0.914)	0.426 (0.227–0.799)	0.373 (0.187–0.745)	0.609 (0.352–1.055)	0.457 (0.196–1.066)	0.359 (0.179–0.718)
HDL-C (mg/dL)						
≥ 40	1.000 (reference)	0.839 (0.542–1.299)	1.000 (reference)	0.965 (0.624–1.492)	1.000 (reference)	1.119 (0.683–1.836)
< 40	2.706 (1.500–4.882)	2.259 (1.162–4.394)	2.575 (1.312–5.053)	2.624 (1.442–4.775)	4.639 (1.799–11.961)	2.137 (1.141–4.001)

Upper and lower 15% cut-off values of homocysteine and folate were 13.3 μmol/L and 3.7 ng/mL, respectively

*AOR*, adjusted odds ratio (adjusted for age, gender, hypertension, diabetes mellitus); *CI*, confidence interval

### Associations of miRNA SNPs with CRC survival

**Fig. 2:**
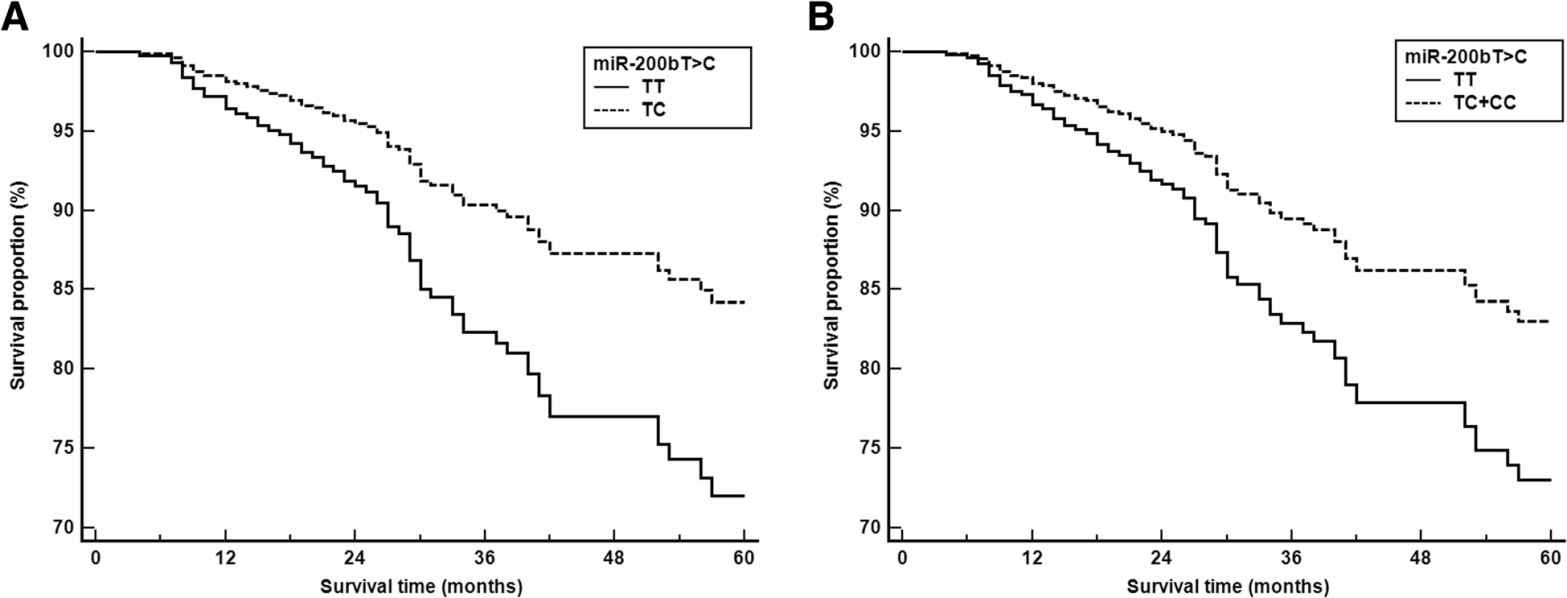
Survival curves depicting the relationship between the *miR-200b*T > C polymorphism and CRC patients. Cox proportional-hazards regression model of CRC patient survival. Patients carrying the *miR-200b* (A) TC and (B) TC + CC genotypes had a reduced risk of death when compared with the TT genotype (*P* = 0.017 and *P* = 0.026, respectively)

Associations between miRNA polymorphisms and CRC survival are shown in Table 5**.** Multivariate Cox proportional analysis showed that the *miR-200b*TC and TC + CC genotypes associated with survival in CRC patients (adjusted HR = 0.522; 95% CI, 0.307–0.888; *P* = 0.017 and adjusted HR = 0.522; 95% CI, 0.307–0.888; *P* = 0.017, respectively; Fig. 2).

**Table 5:** Multivariate survival analysis of polymorphisms in CRC patients

Genotype	CRC(*n* = 472)	Death(*n* = 85)	Adjusted HR^a^(95% CI)	*P*
*miR-130b* rs373001T > C				
TT	269 (57.0)	47 (55.3)	1.000 (reference)	
TC	168 (35.6)	29 (34.1)	0.810 (0.491–1.338)	0.411
CC	35 (7.4)	9 (10.6)	1.345 (0.632–2.864)	0.442
Dominant (TT vs TC + CC)			0.910 (0.575–1.438)	0.685
Recessive (TT + TC vs CC)			1.435 (0.688–2.990)	0.336
*miR-200b* rs7549819T > C				
TT	216 (45.7)	48 (56.5)	1.000 (reference)	
TC	200 (42.4)	26 (30.6)	0.522 (0.307–0.888)	0.017
CC	56 (11.9)	11 (12.9)	0.781 (0.393–1.555)	0.482
Dominant (TT vs TC + CC)			0.592 (0.373–0.940)	0.026
Recessive (TT + TC vs CC)			0.994 (0.509–1.944)	0.987
*miR-495* rs2281611A > C				
AA	125 (26.5)	23 (27.1)	1.000 (reference)	
AC	222 (47.0)	37 (43.5)	1.077 (0.618–1.879)	0.794
CC	125 (26.5)	25 (29.4)	1.167 (0.628–2.170)	0.625
Dominant (AA vs AC + CC)			1.126 (0.672–1.886)	0.652
Recessive (AA+AC vs CC)			1.147 (0.691–1.903)	0.595

^**a**^HR estimates with 95% CI and *P*-values from the Cox-proportional hazard model on overall survival. *HR*, hazard ratio (adjusted for age, gender, chemotherapy, TNM stage); *CI*, confidence interval

## Discussion

In this study, we investigated whether the miRNA polymorphisms *miR-130b*T > C rs373001, *miR-200b*T > C rs7549819, and *miR-495*A > C rs2281611 associate with susceptibility for CRC or a CRC subgroup in Korean subjects. These three SNPs are regulatory SNPs located in the promoter regions of the miRNA genes. SNPs in the promoter regions of miRNAs have been shown to affect the expression of mature miRNAs that regulate target genes [24, 25].

*miR-495* has been shown to play a tumor suppressor role in many cancers, including gastric cancer [37], non-small cell lung cancer [38], glioma [39], and CRC [40]. In particular, *miR-495* has been shown to regulate expression of genes involved in cellular processes, including mTOR, Akt, and PRL-3 [37, 41, 42]. Our data suggest that the *miR-495*CC genotype associates with an increased risk for rectal cancer when compared with the other genotypes**.** Therefore, we assume that substitution of the C allele with the rs2281611 A allele in the promoter region of the *miR-495* gene leads to a reduction in miRNA expression, which then affects CRC susceptibility. In the combinatorial gene-environment analysis, the *miR-495*CC genotype combined with folate exhibited a significantly increased risk of CRC. Folic acid is an essential factor involved in one-carbon metabolism, including DNA synthesis, repair, and methylation [43–45]. When the folate level is insufficient, DNA is abnormally replicated during cell division [46], DNA is degraded, and mutagenesis increases [43]. In addition, uracil misincorporation and double-strand breaks have been observed in tumor cells cultured in low folate conditions [43, 47]. Low folate levels have also been associated with breast cancer [48], CRC [49], and gastric cancer [50]. Thus, the effects of the *miR-49*5CC genotype and low folate concentration appear to be synergistic.

In the survival analysis, the *miR-200b*TC and TC + CC genotypes associated with the survival rate of patients who had undergone CRC resection. The miR-200 family has been shown to inhibit EMT, which shares many similarities with cancer progression [51], and to associate with poor prognoses, including metastasis, invasion, and chemoresistance in gastric cancer [52], bladder cancer [53], and CRC [54]. The miR-200 family has also been implicated in CRC survival [55]. Abnormal miR-200b expression moderates the poor prognosis and progression of CRC, and these factors may affect patient survival rate.

There are several limitations to our study. The first is that expression differences in mature miRNAs due to SNPs in the regulatory regions of miRNA genes have not been confirmed at the molecular and functional levels. Therefore, we are inferring that expression of the altered *miR-495* relates directly to CRC risk by targeting the tumor suppressor gene. The second limitation is that the sample size may be insufficient to draw any conclusions from the stratified analysis. Future studies should include more than 1000 ethnically homogeneous people. Lastly, this study only included Koreans who visited CHA Bundang Medical Center. Although our findings provide the first evidence that miRNA polymorphisms could be potential biomarkers of CRC prevention and prognosis, significant results should be identified in independent populations to confirm the validity of these results.

## Conclusion

In conclusion, we investigated the relationship between CRC susceptibility and the miRNA polymorphisms *miR-130b* rs373001, *miR*-*200b* rs7549819, and *miR*-*495* rs2281611. We found that *miR-200b* and *miR*-*495* associated with CRC susceptibility and survival of CRC patients, respectively. Although there have been many studies that have described the relationships between *miR-200b* and *miR*-*495* and CRC susceptibility, no associations between the *miR-200b* and *miR-495* polymorphisms and CRC have been reported. Thus, our results provide evidence that *miR-200b* and *miR-495* polymorphisms may be potential biomarkers for CRC diagnosis and prevention.

## Additional file

Additional file 1: **Table S1.** Information of *miR-200* and *495* polymorphisms for PCR-RFLP. **Table S2.** Comparison of genotype frequencies of microRNA polymorphisms between colorectal cancer subtype and control. **Table S3.** Stratified effects of *miR-130b*T > C, *miR-200b*T > C, and *miR-495*C > A polymorphisms on CRC susceptibility. **Table S4.** Combinatorial effects of miRNA polymorphisms and environmental factors on CRC risk. (DOCX 26 kb)

## Abbreviations

AJCC: American joint committee on cancer
AOR: Adjusted odds ratio
CI: Confidence interval
CIMP: CpG island methylator phenotype
CIN: Chromosomal instability
CRC: Colorectal cancer
DM: Diabetes mellitus
EMT: Epithelial mesenchymal transition
FDR: False discovery rate
HR: Hazard ratio
HTN: Hypertension
HWE: Hardy-weinberg equilibrium
miRNA: microRNA
MSI: Microsatellite instability
pre-miRNA: precursor miRNA
pri-miRNA: primary miRNA
RISC: RNA-induced silencing complex
SNP: Single nucleotide polymorphism
TNM: Tumor, node and metastasis classification
UTR: Untranslated region
