# Supplementary material for: Genetic associations between the miRNA polymorphisms miR-130b (rs373001), miR-200b (rs7549819), and miR-495 (rs2281611) and colorectal cancer susceptibility
Source: BMC Cancer. 2019 May 22;19:480. doi: 10.1186/s12885-019-5641-1 (PMC6532172; doi:10.1186/s12885-019-5641-1)
Supplement: Supplementary file 1 — Table S1. Information of miR-200 and 495 polymorphisms for PCR-RFLP. Table S2. Comparison of genotype frequencies of microRNA polymorphisms between colorectal cancer subtype and control. Table S3. Stratified effects of miR-130bT > C, miR-200bT > C, and miR-495C > A polymorphisms on CRC susceptibility. Table S4. Combinatorial effects of miRNA polymorphisms and environmental factors on CRC risk. (DOCX 26 kb) [file 12885_2019_5641_MOESM1_ESM.docx]

| **Table S1** Information of *miR-200, and 495* polymorphisms for PCR-RFLP. | | | |  |  |  |
| --- | --- | --- | --- | --- | --- | --- |
| Genotype | Reference SNP ID | Primer sequence | | annealing condition | Restriction enzyme | RFLP condition |
| *miR-200b*T>C | rs7549819 | Forward : | 5’- CCT GAA CCT GGC AGT GG -3’ | 60°C , with 35 cycles | AciI | incubation for  16 h at 37°C |
|  |  | Reverse : | 5’- CAG TGC TTC AGG AAC ACA ATT T -3’ |  |  |  |
| *miR-495*A>C | rs2281611 | Forward : | 5’- GCA TCA GGT AAG TTG GGT CA -3’ |  | HincII |  |
|  |  | Reverse : | 5’- TTA TCC GTG ATG ACT GTC CG -3’ |  |  |  |

| **Table S2 Comparison of genotype frequencies of microRNA polymorphisms between colorectal cancer subtype and control.** | | | | | | | | | | | | | |
| --- | --- | --- | --- | --- | --- | --- | --- | --- | --- | --- | --- | --- | --- |
| **Genotypes** | **Controls (n=399)** | **MSS (n=329)** | **AOR (95% CI)** | ***P*** | **MSI (n=61)** | **AOR (95% CI)** | ***P*** | **MSI-High (n=46)** | **AOR (95% CI)** | ***P*** | **MSI-Low (n=15)** | **AOR (95% CI)** | ***P*** |
| ***miR-130b* rs373001T>C** |  |  |  |  |  |  |  |  |  |  |  |  |  |
| TT | 216 (54.2) | 193 (58.7) | 1.000 (reference) |  | 37 (60.7) | 1.000 (reference) |  | 27 (58.7) | 1.000 (reference) |  | 10 (66.7) | 1.000 (reference) |  |
| TC | 157 (39.3) | 115 (35.0) | 0.845 (0.607 - 1.177) | 0.319 | 20 (32.8) | 0.662 (0.362 - 1.210) | 0.180 | 16 (34.8) | 0.727 (0.368 - 1.434) | 0.358 | 4 (26.7) | 0.503 (0.153 - 1.653) | 0.257 |
| CC | 26 (6.5) | 21 (6.4) | 0.875 (0.459 - 1.668) | 0.685 | 4 (6.6) | 0.803 (0.259 - 2.492) | 0.704 | 3 (6.5) | 0.799 (0.220 - 2.908) | 0.734 | 1 (6.7) | 0.866 (0.104 - 7.178) | 0.894 |
| Dominant (TT vs TC+CC) |  |  | 0.852 (0.622 - 1.168) | 0.320 |  | 0.679 (0.384 - 1.200) | 0.183 |  | 0.728 (0.382 - 1.389) | 0.336 |  | 0.552 (0.183 - 1.663) | 0.291 |
| Recessive (TT+TC vs CC) |  |  | 0.952 (0.509 - 1.779) | 0.877 |  | 0.898 (0.294 - 2.745) | 0.850 |  | 0.850 (0.237 - 3.043) | 0.803 |  | 1.055 (0.132 - 8.464) | 0.960 |
| ***miR-200b* rs7549819T>C** |  |  |  |  |  |  |  |  |  |  |  |  |  |
| TT | 171 (42.9) | 146 (44.4) | 1.000 (reference) |  | 24 (39.3) | 1.000 (reference) |  | 18 (39.1) | 1.000 (reference) |  | 6 (40.0) | 1.000 (reference) |  |
| TC | 176 (44.1) | 145 (44.1) | 0.955 (0.685 - 1.331) | 0.786 | 29 (47.5) | 1.196 (0.661 - 2.163) | 0.554 | 22 (47.8) | 1.252 (0.638 - 2.458) | 0.513 | 7 (46.7) | 1.052 (0.339 - 3.264) | 0.930 |
| CC | 52 (13.0) | 38 (11.6) | 0.765 (0.462 - 1.268) | 0.299 | 8 (13.1) | 0.983 (0.394 - 2.451) | 0.971 | 6 (13.0) | 1.013 (0.351 - 2.927) | 0.981 | 2 (13.3) | 1.049 (0.203 - 5.432) | 0.954 |
| Dominant (TT vs TC+CC) |  |  | 0.914 (0.668 - 1.251) | 0.575 |  | 1.154 (0.656 - 2.031) | 0.619 |  | 1.202 (0.632 - 2.285) | 0.574 |  | 1.083 (0.372 - 3.155) | 0.884 |
| Recessive (TT+TC vs CC) |  |  | 0.775 (0.479 - 1.253) | 0.298 |  | 0.887 (0.378 - 2.085) | 0.784 |  | 0.896 (0.333 - 2.410) | 0.828 |  | 0.959 (0.207 - 4.436) | 0.957 |
| ***miR-495* rs2281611A>C** |  |  |  |  |  |  |  |  |  |  |  |  |  |
| AA | 103 (25.8) | 94 (28.6) | 1.000 (reference) |  | 13 (21.3) | 1.000 (reference) |  | 13 (28.3) | 1.000 (reference) |  | 0 (0.0) | 1.000 (reference) |  |
| AC | 194 (48.6) | 149 (45.3) | 0.758 (0.517 - 1.110) | 0.155 | 31 (50.8) | 1.109 (0.541 - 2.270) | 0.778 | 20 (43.5) | 0.689 (0.318 - 1.494) | 0.346 | 11 (73.3) | N/A | 0.998 |
| CC | 102 (25.6) | 86 (26.1) | 0.985 (0.646 - 1.501) | 0.943 | 17 (27.9) | 1.328 (0.608 - 2.902) | 0.478 | 13 (28.3) | 1.005 (0.438 - 2.306) | 0.991 | 4 (26.7) | N/A | 0.998 |
| Dominant (AA vs AC+CC) |  |  | 0.842 (0.594 - 1.195) | 0.336 |  | 1.210 (0.622 - 2.356) | 0.575 |  | 0.822 (0.409 - 1.652) | 0.582 |  | N/A | 0.998 |
| Recessive (AA+AC vs CC) |  |  | 1.182 (0.830 - 1.683) | 0.353 |  | 1.187 (0.641 - 2.196) | 0.586 |  | 1.216 (0.607 - 2.438) | 0.581 |  | 1.053 (0.326 - 3.409) | 0.931 |
| AOR, adjusted odds ratio (adjusted for age, gender, hypertension, diabetes mellitus); CI, confidence interval; MSS, microsatellite stable; MSI, microsatellite instability. | | | | | | | | | | | | | |

| **Table S3 Stratified effects of *miR-130b*T>C, *miR-200b*T>C, and *miR-495*C>A polymorphisms on CRC susceptibility.** | | | | | | |
| --- | --- | --- | --- | --- | --- | --- |
| **Variables** | ***miR-130b* TT vs TC+CC** | | ***miR-200b* TT vs TC+CC** | | ***miR-495* AA+AC vs CC** | |
|  | **AOR (95% CI)** | ***P*** | **AOR (95% CI)** | ***P*** | **AOR (95% CI)** | ***P*** |
| **Age(year)** |  |  |  |  |  |  |
| <64 | 0.812 (0.546 - 1.207) | 0.303 | 0.742 (0.499 - 1.101) | 0.138 | 1.105 (0.700 - 1.745) | 0.668 |
| ≥64 | 0.881 (0.575 - 1.350) | 0.561 | 0.900 (0.583 - 1.390) | 0.635 | 1.353 (0.849 - 2.158) | 0.204 |
| **Gender** |  |  |  |  |  |  |
| Male | 0.995 (0.641 - 1.544) | 0.982 | 0.918 (0.596 - 1.413) | 0.698 | 1.194 (0.729 - 1.955) | 0.480 |
| Female | 0.755 (0.515 - 1.106) | 0.149 | 0.829 (0.563 - 1.222) | 0.343 | 1.248 (0.814 - 1.913) | 0.310 |
| **Hypertension** |  |  |  |  |  |  |
| non-HTN | 0.872 (0.576 - 1.321) | 0.518 | 0.620 (0.409 - 0.940) | 0.024 | 1.452 (0.920 - 2.294) | 0.109 |
| HTN | 0.806 (0.537 - 1.209) | 0.297 | 0.991 (0.656 - 1.499) | 0.967 | 0.996 (0.631 - 1.572) | 0.986 |
| **Diabetes mellitus** |  |  |  |  |  |  |
| non-DM | 0.829 (0.600 - 1.146) | 0.257 | 0.764 (0.553 - 1.054) | 0.101 | 1.298 (0.911 - 1.849) | 0.149 |
| DM | 0.900 (0.477 - 1.697) | 0.744 | 1.316 (0.696 - 2.487) | 0.399 | 0.965 (0.452 - 2.060) | 0.926 |
| **Homocysteine (μmol/L)** |  |  |  |  |  |  |
| <13.3 | 0.910 (0.655 - 1.263) | 0.571 | 1.151 (0.827 - 1.602) | 0.406 | 1.296 (0.901 - 1.865) | 0.162 |
| ≥13.3 | 0.730 (0.340 - 1.565) | 0.418 | 0.524 (0.241 - 1.137) | 0.102 | 1.249 (0.511 - 3.056) | 0.626 |
| **Folate (nmol/L)** |  |  |  |  |  |  |
| >3.7 | 0.921 (0.663 - 1.278) | 0.623 | 1.030 (0.740 - 1.435) | 0.860 | 1.188 (0.822 - 1.716) | 0.359 |
| ≤3.7 | 0.606 (0.251 - 1.465) | 0.266 | 0.851 (0.344 - 2.110) | 0.728 | 1.224 (0.484 - 3.096) | 0.670 |
| **Triglyceride (mg/dL)** |  |  |  |  |  |  |
| <150 | 0.888 (0.617 - 1.279) | 0.523 | 0.907 (0.629 - 1.308) | 0.602 | 1.372 (0.919 - 2.047) | 0.122 |
| ≥150 | 0.735 (0.416 - 1.297) | 0.288 | 0.998 (0.570 - 1.745) | 0.993 | 1.089 (0.566 - 2.097) | 0.798 |
| **HDL-C (mg/dL)** |  |  |  |  |  |  |
| ≥40 | 1.286 (0.754 - 2.195) | 0.356 | 0.687 (0.399 - 1.184) | 0.177 | 1.066 (0.605 - 1.878) | 0.826 |
| <40 | 0.848 (0.441 - 1.633) | 0.622 | 0.811 (0.424 - 1.551) | 0.526 | 1.228 (0.568 - 2.656) | 0.602 |
| Note. AOR, Adjusted Odds Ratio (adjusted by age, gender, hypertension, diabetes mellitus); CI, confidence interval; HDL-C, High density lipoprotein cholesterol | | | | | | |

| **Table S4 Combinatorial effects of miRNA polymorphisms and environmental factors on CRC risk.** | | | | | | |
| --- | --- | --- | --- | --- | --- | --- |
| **Characteristics** | **miR-130b TT** | **miR-130b TC+CC** | **miR-200b TT** | **miR-200b TC+CC** | **miR-495 AA+AC** | **miR-495 CC** |
|  | **AOR (95% CI)** | **AOR (95% CI)** | **AOR (95% CI)** | **AOR (95% CI)** | **AOR (95% CI)** | **AOR (95% CI)** |
| **Age** |  |  |  |  |  |  |
| <63years | 1.000 (reference) | 0.812 (0.546 - 1.207) | 1.000 (reference) | 0.742 (0.499 - 1.101) | 1.000 (reference) | 1.105 (0.700 - 1.745) |
| ≥63years | 0.895 (0.606 - 1.323) | 0.767 (0.503 - 1.169) | 0.858 (0.563 - 1.307) | 0.824 (0.546 - 1.245) | 0.851 (0.603 - 1.201) | 1.192 (0.770 - 1.847) |
| **Gender** |  |  |  |  |  |  |
| Male | 1.000 (reference) | 0.784 (0.581 - 1.058) | 1.000 (reference) | 0.814 (0.603 - 1.101) | 1.000 (reference) | 1.194 (0.729 - 1.955) |
| Female | 0.899 (0.507 - 1.593) | 1.617 (0.696 - 3.757) | 0.961 (0.494 - 1.870) | 1.209 (0.636 - 2.297) | 0.929 (0.664 - 1.301) | 1.133 (0.729 - 1.761) |
| **Hypertension** |  |  |  |  |  |  |
| No | 1.000 (reference) | 0.852 (0.563 - 1.290) | 1.000 (reference) | 0.629 (0.416 - 0.951) | 1.000 (reference) | 1.428 (0.905 - 2.254) |
| Yes | 2.509 (1.678 - 3.751) | 1.931 (1.256 - 2.969) | 1.801 (1.175 - 2.762) | 1.997 (1.307 - 3.052) | 2.536 (1.782 - 3.610) | 2.904 (1.780 - 4.738) |
| **Diabetes mellitus** |  |  |  |  |  |  |
| No | 1.000 (reference) | 0.824 (0.597 - 1.138) | 1.000 (reference) | 0.754 (0.547 - 1.039) | 1.000 (reference) | 1.277 (0.897 - 1.820) |
| Yes | 2.957 (1.791 - 4.882) | 2.590 (1.559 - 4.304) | 2.319 (1.367 - 3.935) | 2.990 (1.808 - 4.945) | 3.245 (2.144 - 4.911) | 3.096 (1.532 - 6.257) |
| **Homocysteine (μmol/L)** |  |  |  |  |  |  |
| <13.3 | 1.000 (reference) | 0.861 (0.631 - 1.175) | 1.000 (reference) | 0.920 (0.675 - 1.255) | 1.000 (reference) | 1.236 (0.874 - 1.749) |
| ≥13.3 | 1.364 (0.781 - 2.384) | 0.907 (0.485 - 1.698) | 1.761 (0.929 - 3.338) | 0.894 (0.514 - 1.554) | 1.201 (0.737 - 1.958) | 1.428 (0.639 - 3.189) |
| **Folate (nmol/L)** |  |  |  |  |  |  |
| >3.7 | 1.000 (reference) | 0.874 (0.642 - 1.190) | 1.000 (reference) | 0.855 (0.628 - 1.166) | 1.000 (reference) | 1.178 (0.831 - 1.672) |
| ≤3.7 | 2.934 (1.609 - 5.353) | 2.057 (1.031 - 4.102) | 2.557 (1.304 - 5.014) | 2.217 (1.211 - 4.059) | 2.556 (1.476 - 4.426) | 3.119 (1.432 - 6.791) |
| **FBS(mg/dL)** |  |  |  |  |  |  |
| <100 | 1.000 (reference) | 0.897 (0.564 - 1.427) | 1.000 (reference) | 0.730 (0.459 - 1.162) | 1.000 (reference) | 1.784 (1.079 - 2.951) |
| ≥100 | 1.231 (0.818 - 1.851) | 0.971 (0.623 - 1.514) | 1.108 (0.706 - 1.739) | 0.927 (0.598 - 1.438) | 1.423 (0.984 - 2.057) | 1.495 (0.934 - 2.392) |
| **Metabolic syndrome** |  |  |  |  |  |  |
| No | 1.000 (reference) | 0.831 (0.609 - 1.134) | 1.000 (reference) | 0.786 (0.576 - 1.072) | 1.000 (reference) | 1.251 (0.886 - 1.765) |
| Yes | 1.319 (0.776 - 2.242) | 1.179 (0.641 - 2.168) | 1.074 (0.586 - 1.968) | 1.323 (0.775 - 2.259) | 1.364 (0.863 - 2.155) | 1.558 (0.701 - 3.464) |
| Upper and lower 15 % cut-off values of homocysteine and folate were 13.3 μmol/L and 3.7 ng/mL, respectively. AOR, adjusted odds ratio (adjusted for age, gender, hypertension, diabetes mellitus); CI, confidence interval | | | | | | |
